# Why People Forgo Healthcare in France: A National Survey of 164 092 Individuals to Inform Healthcare Policy-Makers

**DOI:** 10.34172/ijhpm.2022.6310

**Published:** 2022-06-11

**Authors:** Najeh Daabek, Sébastien Bailly, Alison Foote, Philippe Warin, Renaud Tamisier, Hélèna Revil, Jean-Louis Pépin

**Affiliations:** ^1^HP2 laboratory, INSERM U1300, University Grenoble Alpes, Grenoble, France; ^2^AGIR à Dom, Homecare charity, Grenoble, France; ^3^EFCR Laboratory, Grenoble Alpes University Hospital, Grenoble, France.; ^4^Research Division, Grenoble Alpes University Hospital, Grenoble, France; ^5^Social Sciences Research – PACTE Laboratory, CNRS UMR 5194, University Grenoble Alpes, Grenoble, France.

**Keywords:** Healthcare Forgoers, Renunciation, Non-take-up, Inequality, Survey, France

## Abstract

**Background:** Even in countries having nearly universal healthcare provision some individuals forgo or postpone healthcare to which they are entitled. Socioeconomic and geographic inequalities can make access to healthcare difficult for some people, such that they fail to seek it, particularly if they deem the type of care as non-essential. The need to pay at the point of care, the complexity and cost of top-up health insurance, and delays or only partial reimbursement can discourage take-up of care. This can affect the general health of the population.

**Methods:** To estimate the rate of forgoing healthcare in the general French population, between 2015 and 2018 we conducted a nationwide cross-sectional survey of individuals visiting French primary healthcare insurance agencies (Caisse Primaire d’Assurance Maladie, CPAM). We asked whether the person had foregone or postponed healthcare in the last 12 months, if so the types of healthcare forgone or put-off, and reasons. Individuals were stratified by the type of complementary (top-up) health insurance they had.

**Results:** Out of 164 092 individuals who responded, 158 032 were included in the analysis. Respondents had either private complementary (top-up) insurance (60%), top-up insurance subsidized by the state (29%), or no top-up health insurance (11%). Forgoers (n=40 115; 25.4%) most often lived alone (with or without children), were unemployed, and/ or female. Dental care (54%) and consultations with ophthalmologists, gynaecologists and dermatologists (41%) were most commonly forgone. The reasons were: inability to advance payment and/or to pay the uninsured part (69%), time constraints and difficulty in obtaining appointments (26%).

**Conclusion:** We present a snapshot of forgoing healthcare in a developed country, highlighting the need for continuing review by policy-makers of payment regimens, insurance cover, availability and accessibility. While initiatives have already emerged from the results, further reforms are needed to address the problem of people forgoing preventative or perceived non-urgent healthcare, particularly for disadvantaged subgroups.

## Background

**Key Messages**

** Implications for policy makers**

- To improve fair and equal access to healthcare, modifiable social, geographical and financial determinants of access to healthcare need to be addressed.
- Policies at national, local and individual patient levels need to be reviewed to minimize non take up of care.
- Innovative tools for repeated cross-sectional evaluation of forgoing healthcare and measuring the impact of reforms aimed at providing better health cover and access to services should be deployed internationally.

** Implications for the public**

Not taking-up healthcare to which one is entitled is quite common. This may be for financial reasons such as inability to make any required point-of-care payment, complex bureaucracy to obtain reimbursement, lack of complementary (top-up) insurance and other reasons. However it may also due to mobility and transport problems, long waiting times or no local general practitioner (GP). We surveyed people registered with the French social security system. Already the preliminary results of our survey, and other surveys, have led to the introduction of a simpler system for people on low incomes unable to pay for complementary insurance. In addition, cost-free provision of basic glasses and hearing aids has been implemented.

Most people would agree that healthcare is a universal right upheld by five ethical pillars: universality, fair and equal access, affordability, quality and choice.^1^ Despite the overall high performance of the French healthcare system,^2^ recent studies have highlighted not only socioeconomic and geographic inequalities but also personal behaviours and beliefs that lead some people to forgo healthcare (sometimes called “non-take-up” of care) in France,^3^ Europe^4,5^ and elsewhere.

 The health system (particularly the availability of services, payment at the point of care, and the proportion of costs that are reimbursed), socioeconomic status and even personal conducts and/or beliefs, can lead individuals to forgo or postpone identified healthcare needs. Several reports from different perspectives have all emphasized the importance of dealing with this phenomenon given the adverse impact it may have on the health of the population as a whole and the burden it poses on the healthcare system.^3,6-9^

 Three international surveys carried out in Europe^10,11^ and Commonwealth countries^12^ addressed the phenomenon of unmet needs and included data on forgone healthcare. These surveys presented large disparities in the results obtained due to methodological differences, either in the population studied, the definition of unmet needs, and/or the reasons and the types of healthcare considered.^13^ In France, the CONSTANCES cohort is an ongoing epidemiological research cohort of healthy volunteers focusing on financial reasons for forgoing healthcare, among other health issues.^14,15^

 A large part of research on forgoing healthcare has focused on a particular type of care (for example dental care^7^), specific populations (mainly vulnerable groups: students, migrants, the elderly etc^8,9^) or on certain types of reasons (financial reasons mainly).^7,9,16^ The study focusing on dental care^7^ showed that, although financial reasons were the main raison for forgoing treatment, there were multiple factors involved in the phenomenon and a wider perspective was necessary to account for all people forgoing healthcare when formulating health policies.^7^ It was also shown that low income, a poor general state of health, family situation (particularly for women), unemployment, low self-confidence and work-related constraints are some of the multiple factors associated with financial reasons for forgoing healthcare.^9,16^

 In the present study we attempted to reach a broader more general population, to explore the disparities between the French “départements” (administrative areas equivalent to counties in the United States or United Kingdom and hereafter referred to as county/counties), and to study the determinants of forgoing healthcare for not only financial but also non-financial reasons. We considered the distinctive aspects of the French healthcare system and the French population, using a survey questionnaire we had constructed.

###  Healthcare Cover in France 

 France has a two-tier system of healthcare cover, (*i*) compulsory ‘primary’ health insurance schemes with income-proportional contributions, with access to care determined in accordance with needs and reimbursement of a percentage of the costs; and (*ii*) optional public or private complementary (top-up) health insurance schemes known as “mutuelles” that usually, but not always, reimburse the remaining part of the costs.

 Most people are registered with the local branch of the general primary health insurance scheme “Caisse Primaire d’Assurance Maladie” (CPAM) under the direction of the “Caisse national d’assurance maladie” (CNAM). People visit their local CPAM agency (usually several offices in each county) for administrative health insurance related questions and procedures, but not for medical appointments. People in some specific sectors of activity (agricultural workers, civil servants etc) have parallel compulsory primary health insurance schemes and were not surveyed or included in our analysis.

 The reimbursement of consultations and treatments by the compulsory health insurance is made according to two factors: the reimbursement base which sets the maximum amount that can be covered by compulsory health insurance; and the reimbursement rate, expressed as a percentage, which often limits coverage at 60% to 90% of the reimbursement base. These elements are negotiated with the medical professions at a national level and are the subject of contracts. For drugs, it is the National Union of Health Insurance Funds that sets the reimbursement rate on the basis of the actual medical service rendered and the gravity of the condition concerned.

 Except for individuals with chronic pathologies registered in a national database, compulsory health insurance does not therefore reimburse all care, equipment or medication; the remainder to be paid by the patient is called the “co-payment.” This is particularly high for certain types of care, acts or equipment notably dental prostheses, glasses and hearing aids. Moreover, certain physicians can charge more than the negotiated fees (the object of a contract with CNAM) and the “excess fees” are not covered by the compulsory health insurance (see Supplementary file 1).

 The expenses not reimbursed by compulsory health insurance (co-payment and excess fees) may be covered by complementary (or “top-up”) health insurance, up to the guarantees set in the contracts taken out by the individuals. However, in 2019, 5% of French residents did not have complementary health insurance, often for lack of the financial means to pay for it. This figure is significantly higher in certain categories of the population, in particular low-income people. People not covered by a complementary insurance must therefore pay in full the portion of health costs not reimbursed by compulsory health insurance (ie, co-payment) and any excess fees, usually at the point-of-care. It should be noted that alongside people who do not have any complementary insurance, many people cannot pay for a level of health insurance with sufficient guarantees in relation to their needs and must, while paying for a complementary insurance also finance certain out-of-pocket point-of-care expenses (certain -payment and excess fees).

 In a context where complementary insurance has become essential to limit health costs payable directly by individuals, measures have been put in place by the State so that people whose income does not exceed a certain ceiling can benefit from free complementary health insurance or can be helped to finance their private complementary health insurance.

 The public complementary (top-up) schemes paid for by the State (through a tax on private complementary health insurers) were, until the end of 2019 “complementary universal health coverage” (CMU-C) that provided free complementary health insurance cover for those on a low income, whether unemployed, employed or retired; and “public participation in complementary health insurance” (ACS) that subsidized the cost of private complementary health insurance for people whose resources were low but slightly higher than the ceiling set for the CMU-C. As of 1st November 2019, the CMU-C and the ACS have been merged into a single scheme called “complementary health solidarity” (CSS) to simplify access, limit refusals of care by physicians and other differential treatment of CMU-C beneficiaries and limit the non-take-up (forgoing) by persons eligible for this aid.^17^ Although there is now nominally a single scheme (the CSS), within it there are still two divisions one similar to the CMU-C and the other similar to the ACS. In 2019, 95% of the population had complementary health insurance including 7% benefiting from CMU-C.

 Except for CMU-C and ACS (now the CSS) beneficiaries and in some other situations, healthcare is initially paid for by the patient at the point-of-care and then a percentage of the cost is reimbursed by their compulsory health insurance and the rest (but not necessarily all) by the complementary (top-up) insurer, when people have one. The need to pay straightaway at point-of-care and await reimbursement can be difficult for people on modest incomes with little left over to live on after paying their fixed monthly charges.

 A 2004 health insurance reform aimed in particular at improving the care pathways of patients in the health system and to strengthen the coordination of primary and secondary care, through the requirement for a “referring general practitioner (GP)” (family doctor). Since 2004, everyone must therefore provide the name of their referring GP to their compulsory insurance. In addition, if a person does not follow the coordinated care pathway ie, visit a doctor who is not their referring GP or go directly to consultation with a specialist, the basis for reimbursement of care is reduced.

## Methods

###  Study Design and Study Participants 

 We implemented a national cross-sectional study developed as part of the “Access to healthcare program” led by our laboratory. Data were collected sequentially at 101 local CPAM agencies in 5 successive survey waves over 4 years and 6 months (Supplementary file 2). Subjects who consulted their local CPAM office between January 2014 and June 2018 were requested by the CPAM agent who received them to fill-in a questionnaire (Supplementary file 3). CPAM staff were trained to help people complete the questionnaire.

 The questionnaire was constructed by our laboratory in a qualitative and collaborative investigation process. It aimed at providing knowledge on forgoing healthcare in a population that had not been previously studied, ie, people insured under the French general compulsory health insurance scheme. The questionnaire was based essentially on the results of the survey “What forgoing care means” conducted in 2013 by our laboratory, a team from the French medical research agency (Inserm), a French University Hospital public-health unit, and three Swiss University Hospitals. The questionnaire was also inspired by the way of addressing questions on the non-take-up of care in regular surveys conducted by the French Institute for Research and Documentation in Health Economics and within the framework of the “health, inequalities and social ruptures” cohort. To do this, focus groups including members of our laboratory, CPAM agents and insured volunteers from the Gard county (southern France) co-produced this tool. The level of literacy of individuals likely to forgo healthcare was taken into account.

 The questionnaire was tested in the Gard county by CPAM agents and volunteers insured by the CPAM, who were interviewed. Then adjustments were made taking into account the feedback from all parties, before it was deployed in all French CPAM agencies.

 For the present study, we included people registered with CPAM at the interview date and who answered the key question: “Have you forgone or put-off healthcare on one or more occasions in the last 12 months (yes/no)?” Those who did not reply to this question were not included in the study dataset.

###  Data Collected and Procedures 

####  Forgoing Healthcare Data 

 Data were collected using the census method in which the questionnaire was systematically proposed to all persons attending the CPAM agency during the period of the survey wave.

 Those who agreed to participate and replied to the key question were given the questionnaire containing 24 questions organized into four sections **(**Supplementary file 3):

- *General participant characteristics: *Gender, age-band, socio-professional category and family situation.
- *Health characteristics:* Their perception of their current state of health.
- *Healthcare insurance:* Whether they had complementary (top-up) health insurance, the type of complementary health insurance: private insurance, CMU–C or ACS, and whether they were registered with a GP.
- *Healthcare forgone:* If they answered ‘yes’ to the key question they were asked for details about the type(s) of healthcare forgone, the reason for foregoing or putting it off, how long they had been forgoing or putting-off healthcare and the impact (if any) on their life.

###  Statistical Analyses 

####  Descriptive Analyses and Spatial Distribution of People Forgoing Healthcare

 Only categorical variables were collected, for which rates were calculated. The chi-square test was used for comparisons between groups with different healthcare insurance statuses. A *P* value <.05 was considered as significant.

 Due to the low rate of missing data (<1%), a simple imputation method was used, replacing missing values by the most frequent observation. To visualize the spatial distribution of forgoing healthcare in France, a population-adjusted mean rate was calculated for each county using direct standardization methods^18^ (Supplementary file 2) and presented using the maptools package^19^ of R version 3.5.1 (R Core Team 2019).^20^

####  Individual Determinants of Forgoing Healthcare 

 A mixed logit multinomial regression model with a random effect on geographical location was used to analyse the association between an individual’s personal characteristics (introduced as fixed effects) and the probability of forgoing healthcare for a specific reason. Thus the dependant variable is a categorical variable with three levels describing forgoing healthcare status: (1) Did not forgo healthcare, (2) Forwent healthcare for financial reasons and (3) Forwent healthcare for other reasons. Explanatory variables were considered first individually by univariable analyses and variable selection was then performed to obtain a multivariable model were all risk factors were introduced. The association between each risk factor and forgoing healthcare status was estimated using the odds ratio (OR) and its Wald 95% confidence interval (CI). ORs were interpreted comparatively to the first category which is the reference (Did not forgo healthcare). Illustrative examples are provided in the results. Further details of the statistical analysis method are given in Supplementary file 2. To consider both socio-professional categories and type of complementary health insurance, a categorical variable with 12 modes (listed in the supplement) was created.

 Collinearity between the variables was tested by means of Cramer’s V test. However, as associations between variables were all low to moderate (Cramer V varied from 0.02 to 0.5) no collinearity was considered.

 In addition, we performed a subgroup analysis using simply “working” and “retired or not-working” subgroups, where “not-working” included retired and unemployed participants.

 Statistical analyses were performed using SAS 9.4 software (SAS Institute, Inc., Cary, North Carolina) and R version 3.5.1 (R Core Team 2019).^20^

## Results

###  Study Population

 A total of 164 092 individuals answered the questionnaire during the five waves of the study period, among these 367 (0.2%) did not answer the key question on whether they had either forgone or put-off healthcare in the last 12 months. Finally, 158 032 individuals registered in the general primary health insurance scheme were included in the analysis (people registered in other schemes eg, the compulsory scheme for agricultural workers, were excluded). Among people included 40 115 (25.4%) reported forgoing healthcare in the previous 12 months **(**Supplementary file 4).

 Participants were in the majority female (59.8%), working (48.4%), and living alone (49.4%). Their self-assessed health status was “good” for about half of them (50.2%). A majority of the study population had non-subsidized private complementary (top-up) healthcare insurance (59.6%) and 21.6% had full public complementary health insurance (CMU-C). Approximately 5% were not registered with a referring GP, irrespective of type of complementary insurance (Table).

**Table:** Baseline Characteristics of Individuals Not-forgoing or Forgoing Healthcare in France

**Variable**	**Total Sample ** **N = 158 032 **	**Not Forgoing Care ** **n = 117 917 (74.6%) **	**Forgoing Care** **n = 40 115 (25.4%)**	**Missing (%)**	***P*** **Value**^a^
	**No. (%) **	**No. (%) **	**No. (%) **		
Gender (Female)	93 709 (59.8)	67 505 (57.8)	26 204 (65.8)	0.85	<.0001
Age				0.43	<.0001
Under 24 years	14 152 (9.1)	10 996 (9.3)	3156 (7.9)		
[25-39] years	41 133 (26.1)	30 490 (26)	10 643 (26.6)		
[40-59] years	68 824 (43.7)	49 905 (42.5)	18 919 (47.4)		
≥60 years	33 242 (21.1)	26 011 (22.2)	7231 (18.1)		
Family situation				0.23	<.0001
Living alone	77 916 (49.4)	55 819 (47.4)	22 097 (55.2)		
Living as a couple (with or without children)	76 400 (48.5)	59 247 (50.4)	17 153 (42.9)		
Other	3345 (2.1)	2585 (2.2)	760 (1.9)		
Socio-professional situation				0.22	<.0001
Working	76 277 (48.4)	57 941 (49.3)	18 336 (45.8)		
Not-working	55 964 (35.5)	39 555 (33.6)	16 409 (41.0)		
Retired person	25 397 (16.1)	20 131 (17.1)	5266 (13.2)		
Complementary healthcare insurance				0.07	<.0001
Private complementary insurance	94 139 (59.6)	72 536 (61.6)	21 603 (53.9)		
Public CMU-C	34 072 (21.6)	26 315 (22.3)	7757 (19.4)		
No complementary healthcare insurance	17 606 (11.1)	10 265 (8.7)	7341 (18.3)		
ACS	12 090 (7.7)	8715 (7.4)	3375 (8.4)		
Not registered with a GP	7958 (5.1)	5385 (4.6)	2573 (6.5)	0.83	<.0001
Health perception				0.34	<.0001
Good	79 027 (50.2)	64 114 (54.6)	14 913 (37.3)		
Average	56 929 (36.2)	39 938 (34)	16 991 (42.5)		
Poor	21 523 (13.7)	13 439 (11.4)	8084 (20.2)		

Abbreviations: CMU-C, complementary universal health coverage; ACS, public participation in complementary health insurance; GP, general practitioner. ^a^*P* values <.05 were considered as significant.

###  Complementary Healthcare Insurance Status and Forgoing Healthcare 

 Healthcare forgoers, compared to non-forgoers, were more likely to be female (65.8% vs 57.8%, *P* < .01), out-of-work (41% vs 33.6%, *P* < .01) living alone (55.2% vs 47.4%, *P* < .01), and/or without any complementary health insurance (18.3% vs 8.7%, *P* < .01). The majority of forgoers had a middling perception of their health, neither good nor bad, (42.5% vs 34% for the non-forgoers, *P* < .01) (Table).

###  Pattern of Forgoing Healthcare Across France 

**Figure 1:**
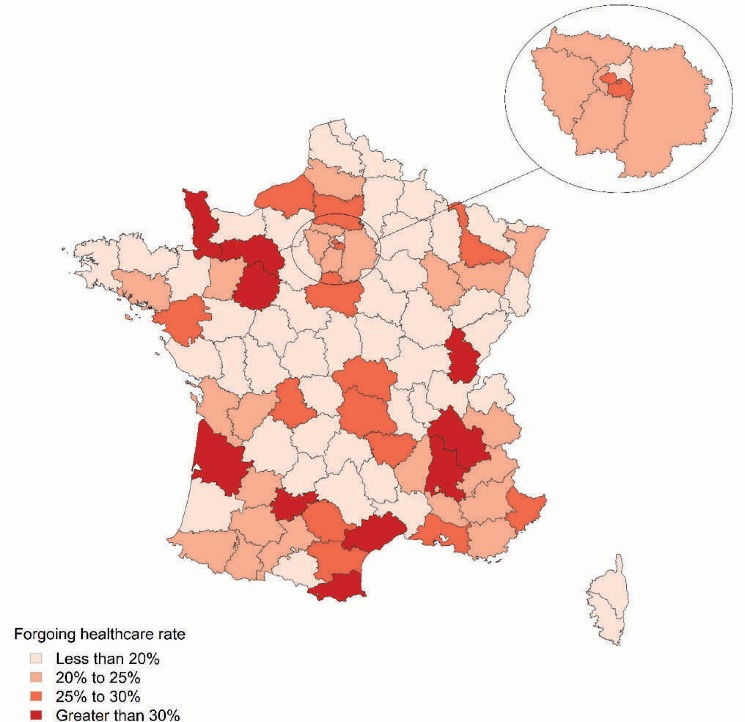
Distribution of Population-Adjusted Mean Rate of Forgoing Healthcare Among the French Counties. The inset shows the Paris agglomeration (Ile-de-France region).

 The population-adjusted mean rate of forgoing healthcare ranged from 10% in the county of Indre (in central France) to 38% in the county of Jura (in eastern France). Large discrepancies were noted between the different regions of France (administrative areas about the size of a US state). A high population-adjusted mean rate of forgoing healthcare (greater than 30%) was observed in two southern regions (Occitanie and Auvergne-Rhône Alpes) and in the north (Normandy) **(**Figure 1).

 Although we had initially aimed to perform a detailed spatial analysis of the determinants of forgoing healthcare, this finally proved unsatisfactory. Unfortunately, information from the French National Institute for Statistic and Economic Studies was only available at the county level which does not distinguish urban and rural areas or between different districts in an urban agglomeration. For this reason spatial analysis of determinants is not presented and only discussed in Supplementary file 5. Figure 1 only provides a low resolution pattern of the phenomenon.

###  Types of Healthcare Forgone 

**Figure 2:**
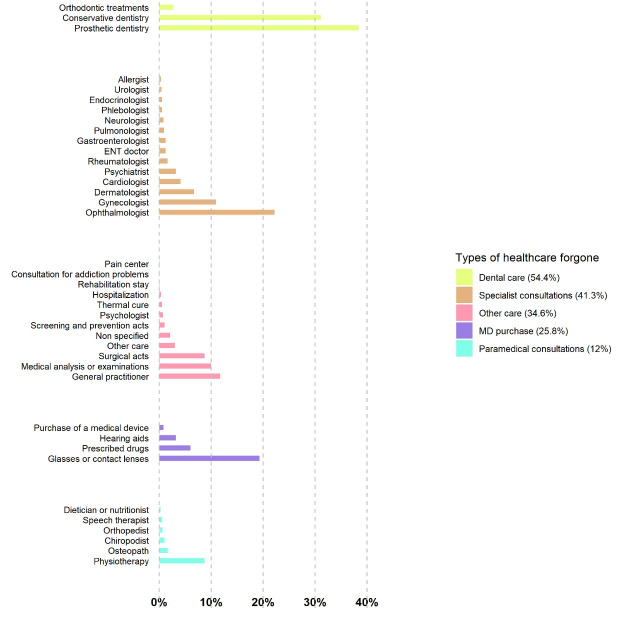
Type of Healthcare Forgone.

 The types of healthcare that subjects most frequently reported forgoing were dental care (54.4%), particularly prosthetic dentistry (38.4%), followed by specialist consultations (41.3%), notably ophthalmic consultations (22.2%), and then consultations with gynaecologists and dermatologists. The other types of forgone healthcare were the purchase of medical devices (25.8%) with glasses and contact lenses at the top of the list (19.3%); followed by consultations with a GP (11.7%), medical laboratory analyses (10%) and physiotherapy (8.7%) (Figure 2).

###  Reasons for Forgoing Healthcare 

 Financial reasons were most frequently given to justify forgoing healthcare (68.9%), where the majority considered that the part of the cost chargeable to the patient was too expensive (60%) even taking into account the contributions of their primary and complementary insurance schemes; or they were unable to advance all or part of payment (30.9%) before receiving reimbursement (despite a third-party payment mechanism).

**Figure 3:**
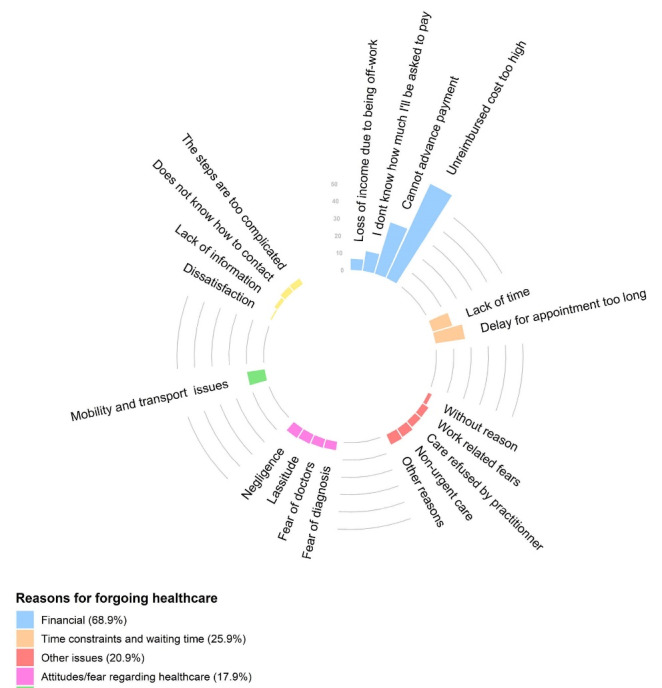
Reasons for Forgoing Healthcare.

 Problems of the individual’s time constraints and appointment availability were the next most frequent reasons for forgoing healthcare (25.9%). Mobility and transport issues were also commonly given as reasons (10%) (Figure 3).

###  Individual Determinants of Forgoing Healthcare 

####  Forgoing Healthcare for Financial Reasons 

**Figure 4:**
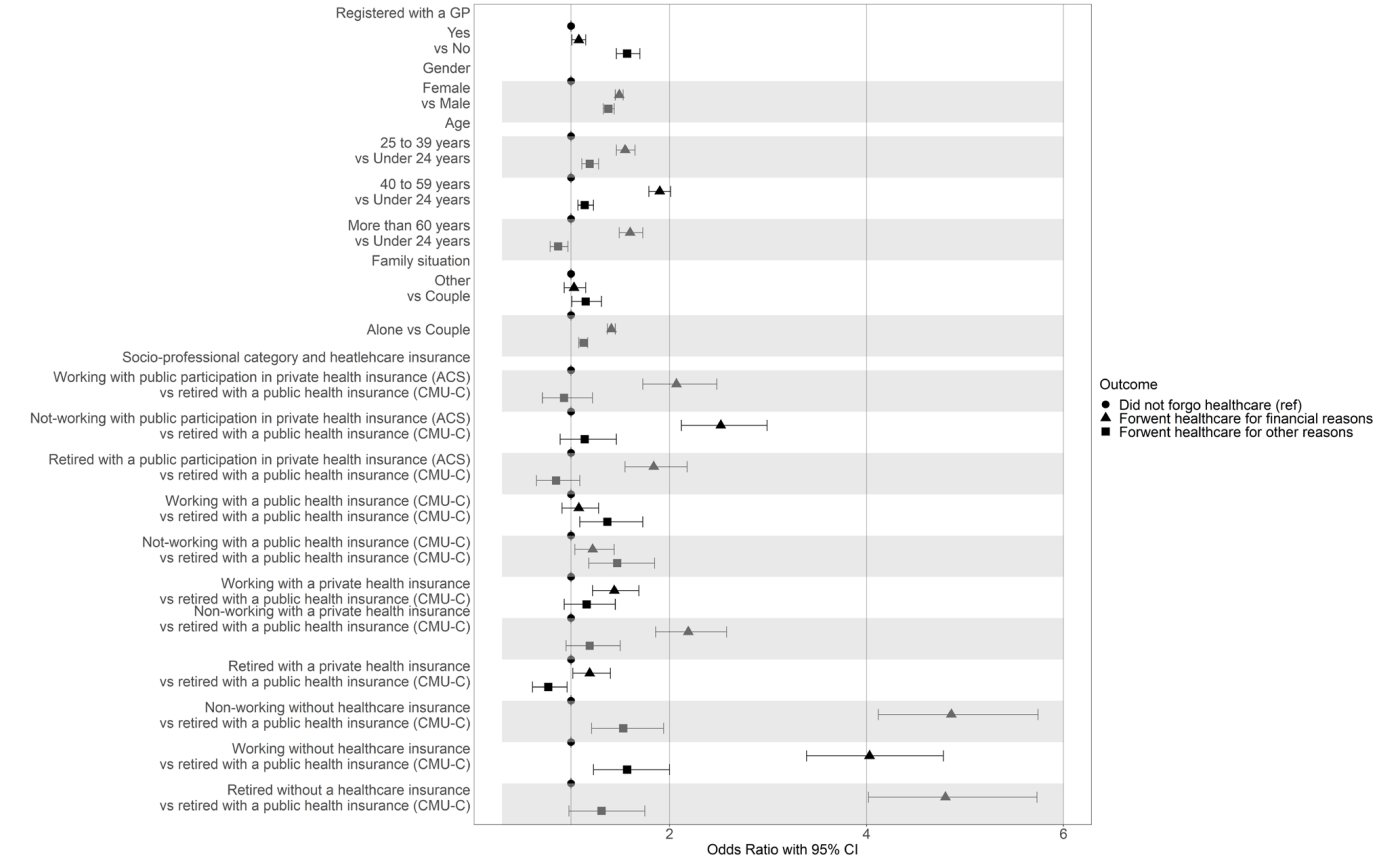
Result of the Multivariable Analysis for Association Between Patient Characteristics and Probability of Forgoing Healthcare. Abbreviations: CMU-C, complementary universal health coverage; ACS, public participation in complementary health insurance; GP, general practitioner; CI, confidence interval.

 Being female, age, family situation, socio-professional category, type of complementary health insurance and not being registered with a GP, were all associated with the probability of forgoing healthcare for financial reasons (Figure 4).

 The factors that increased the likelihood of forgoing healthcare for financial reasons included being over 60 (compared to subjects under 24) (OR: 1.60; 95% CI: 1.49, 1.73); living alone compared to those living in a couple (OR: 1.41; 95% CI: 1.37, 1.45); and to a lesser extent not having a referring GP (OR: 1.08; 95% CI: 1.01, 1.15) (Figure 4).

 Forgoing healthcare for financial reasons was strongly associated with the combination of socio-professional category and having or not having complementary healthcare insurance and whether it was state subsidized. In all socio-professional categories the probability of forgoing healthcare for financial reasons increased by four for people without any complementary health insurance compared to retired persons with full public complementary healthcare insurance (retired CMU-C): if non-working (OR: 4.86; 95% CI: 4.12; 5.74); if working (OR: 4.03; 95% CI: 3.39, 4.78); and if retired (OR: 4.80; 95% CI: 4.02, 5.73). The probability of forgoing healthcare for financial reasons was reduced but not eliminated for people with state subsidized private complementary insurance (ACS): if working (OR: 2.07; 95% CI: 1.73; 2.48); if not working (OR: 2.52%; 95% CI: 2.12, 2.99); and if retired (OR 1.84; 95% CI: 1.55, 2.18). For the retired with private complementary healthcare insurance the probability was slightly higher than for the retired CMU-C group: (OR: 1.19; 95% CI: 1.02; 1.40), but not as high as that for workers (OR: 1.44; 95% CI: 1.22; 1.69) and non-workers (OR: 2.19; 95% CI: 1.86, 2.58). Finally, forgoing healthcare for financial reasons was more likely in non-workers benefiting from full public complementary insurance (CMU-C) (OR: 1.22; 95% CI: 1.04, 1.44), than the retired (Figure 4).

####  Forgoing Healthcare for Other Reasons 

 The probability of forgoing healthcare for reasons other than financial ones was greater for females than for males (OR: 1.38; 95% CI: 1.33, 1.44) and for people living alone (OR: 1.13; 95% CI: 1.08, 1.14) (Figure 4). Compared to people under 24, the over-sixties were less likely to forgo healthcare for non-financial reasons) (OR: 0.87; 95% CI: 0.79, 0.97).

 We observed similar effects of the combination: socio-professional category and type of complementary healthcare insurance on the probability of forgoing healthcare for other reasons than financial ones (working with CMU-C, OR: 1.37, 95% CI: 1.09, 1.73), either non-working or working without any complementary insurance: (respectively OR: 1.53, 95% CI: 1.21; 1.94 and OR: 1.57, 95% CI: 1.23; 2.00) all versus retirees with CMU-C; The exception was the retired with private insurance, where there was a decrease in the probability of forgoing healthcare for other reasons (OR: 0.77, 95% CI: 0.61; 0.96) (Figure 4).

 The results of the binary subgroup analysis “working” vs “retired or not-working” subgroups (see Supplementary file 4) were similar to results given above in which we had distinguished retired and unemployed groups.

## Discussion

 This study allowed us to make an overall assessment of the characteristics of people who forgo healthcare, and their reasons, as well as to highlight the regions of France and types of healthcare that are most affected by the forgoing healthcare phenomenon. The prevalence of people who forwent healthcare in our study population was 25.4% in metropolitan France (ie, not including French overseas territories), which confirms that it is a major societal problem that needs to be addressed by healthcare policy-makers. This rate is considerably higher than the 14% found in the French CONSTANCES study, a much larger broader ranging epidemiological study^15^; probably due to differences in the populations surveyed. The use of CPAM agencies might have biased our population towards people with health insurance difficulties, whereas CONSTANCES recruited healthy volunteers. However, all things being equal, our rate is close to that found in Central and Eastern Europe.^21,22^ Large disparities in the population-adjusted mean rate of all-cause forgoing of healthcare were found among the French counties. Specific types of healthcare were more frequently forgone than others (eg, dental maintenance) and the reasons given for forgoing healthcare were diverse. Given the multi-dimensionality of this phenomenon, the disparities reflect many other underlying territorial inequalities, whether economic, informational, cultural or due to geographic or temporal accessibility, as highlighted in other studies.^23^

 As shown in previous studies,^7^ the most common types of healthcare forgone were specialist care, in particular dental care, ophthalmological care, preventative medicine and diagnostic analyses. This can be explained by the financial structure of the French healthcare insurance system,^17^ and above all by increases in the cost to patients (co-payment and excess fees) for these types of care over the years, through the recovery plans for health insurance, and also by the geographical distribution of the supply of care. This was confirmed by the reasons given for forgoing healthcare: mainly financial and availability ie, the service is not available at all close by, it is available close by but the only appointment possibility is far away in time or not at a time of the day/week when the individual can attend.

 In line with the literature,^8,9^ our study highlighted the fact that having state funded complementary healthcare insurance (CMU-C) or having partial funding by the state toward the cost of private complementary healthcare insurance (ACS), were strong determinants of forgoing healthcare for financial reasons. With the reductions in coverage for routine care of compulsory primary health insurance schemes complementary healthcare insurance has become indispensable. However, a part of the population does not have complementary insurance because they cannot afford the cost. People on low wages (or pensions) and the unemployed have no disposable income after paying fixed charges (rent, electricity, transport, hygiene and food) each month. Moreover, the ACS scheme provided insurance contracts that did not cover all expenses related to care.^23^

 Our large database enabled us to describe the complexity of factors underlying why people forgo healthcare. A simplistic view would have been to consider that financial reasons are the most prominent issues by far overwhelming all other causes. A consequence of such an oversimplification would be to underestimate significant concerns related to the geographical distribution of access to care and transport issues, difficulties in obtaining appointments with practitioners in certain medical specialities and the importance of individual psychosocial behaviours and health beliefs.^24^ Our data demonstrate that forgoing healthcare may be limited to certain particular types of care, in particular dental, ophthalmic, and gynaecologic, notably specialities where preventive care in the form of routine check-ups are usually recommended. Forgoing such types of care could be explained if patients give the care low priority when they do not actually feel ill and by frustration due to access difficulties (such as not being able to obtain an appointment). Qualitative surveys conducted in the “Access to healthcare program,” (Odenore/PACTE) showed that the phenomenon can also be explained by the complexity of the health system. Respondents said they were lost in this system and needed help in navigating it. This has led health insurers to set up platforms to assist in access to healthcare.^25^ A recent step towards simplifying the system has been the replacement of the CMU-C and ACS by the by a single scheme, the “complémentaire santé solidaire” (CSS).

 For a given individual, it is important for social services and their GP to identify factors impeding the take-up of healthcare that can be addressed early-on. In routine general practice, hospitals and clinics simple questionnaires addressing the modifiable risk factors associated with forgoing care could assist clinicians in anticipating non-take up and adherence issues and whether to alert social services.^26-29^ Identifying these factors is probably as important as medication adherence programs. If the reasons are financial, social services can provide help in navigating administrative pathways to access subventions or loans. Also, negative health experiences and patients’ doubts about care efficacy may need the intervention of counselling specialists.

 In a territorial context, our data demonstrate that some concerns are found nationwide, namely difficulties regarding access to dental and ophthalmologic care. This can be addressed by national policies such as the recently implemented universal full reimbursement (“Reste à charge 0”) of dental care and glasses announced by President Macron in 2019. Also some particular populations, such as women living alone on very low incomes, should be the target of national programs. A typical example of postponing care in this group is the delay in screening for breast and uterine cancer leading to unacceptable disparity in outcomes.

 In the United States differences in healthcare up-take between rural and urban regions have been observed.^30^ However, our analysis at a county level didn’t have sufficient resolution to separate cities from rural areas in the same county. Nevertheless, the differences among the French counties in forgoing care call for national measures to facilitate a better territorial repartition of some specific physician workforces as well as a review of policies at the regional and county level.

 To address insufficient physician workforces, digital health and physician assistants may constitute an alternative approach to reducing forgoing healthcare.^31-33^ There is an evolving definition of the GP/primary care provider towards both their partial replacement by a network of supervised physician assistants and e-health supported by artificial intelligence. A challenge will be the capability of health systems to preserve fair and equal access to healthcare when deploying such new forms of organization.

 The majority of the factors we identified that could be used to predict forgoing healthcare are certainly also present in other high-income countries with similar healthcare systems, but with a different ranking depending on the subtleties of national healthcare organization. This field needs new tools to analyse international databases in order to identify the clusters of individuals, behavioural, societal and health system factors that lead to a risk of forgoing or postponing care. Surveys such as ours will constitute a backbone to evaluate the global burden of the problem, inform policy-makers, measure quality of care and suggest improvements in the middle and long term. Innovative tools for sequential cross-sectional evaluation of the extent of forgoing healthcare and measuring the impact of reforms in health systems are needed and should be deployed internationally.

 This study had several limitations leading to biases. First, the questionnaire was only given to individuals who visited their local CPAM agency in person (whatever the reason) rather than consulting the CPAM site (https://www.ameli.fr/), and were interviewed by a member of staff; such people may be more likely to have health/health insurance problems than others. Secondly, some relevant and useful items were not included in the questionnaire and should be included in future studies, including: adults living alone but with or without children, the level of health literacy, and whether the person had recently changed their health insurance cover. Due to the procedures used in administering the questionnaire, we had no information about how many people visited each CPAM agency during the study period and how many refused the questionnaire, thus we cannot exclude a selection bias. Another selection bias was probably the tendency to focus on people with health insurance difficulties, as one would be likely visit a CPAM agency only if there was some problem. The study did not take into account the “social emergency” population, ie, the most vulnerable (people on the street, migrants and refugees, people coming out of prison etc)^34^; which might have led us to underestimate the problem. In France, to avoid discrimination, by law it is not permitted to ask people completing questionnaires/surveys about their ethnicity; data that might have provided additional information and constituted an integrated marker for risk of forgoing healthcare.^35,36^ We used the under-24 group as the age-band comparator, however until September 2018 students had a separate compulsory primary health insurance scheme, thus, our under 24 age-band may have been misrepresented. Using data from the French National Institute for Statistic and Economic Studies we had attempted to analyse the spatial distribution of contextual determinants, but the models were poor and results inconclusive (Supplementary file 5). For medical confidentiality reasons we did not attempt to confront the phenomenon of forgoing healthcare and its consequences with objective measures of the medical condition of the participants. This should be a focus in future studies.

## Conclusion

 Our data describe one consequence, people forgoing healthcare, which results from the interplay between health insurance in France, the French healthcare system, and socioeconomics. We identify specific populations that should be prioritized by healthcare policy-makers. To improve health outcomes and equality in healthcare, modifiable social, geographical and financial determinants of health, particularly those driving people to forgo healthcare, need to be addressed at all levels: national, county, community and individual patient.

## Ethical issues

 This work is based on a survey completed by individuals attending French Health Insurance (CPAM) agencies. In agreeing to participate in the survey and as part of the written informed consent each individual was informed that their anonymized information would be used for research purposes only. They were free to refuse to participate.

## Competing interests

 Outside the context of the submitted work: RT reports other from Agiradom (Healthcare provider), grants from Resmed. JLP reports grants from Air Liquide Foundation, grants and personal fees from Agiradom, grants and personal fees from AstraZeneca, grants from Fisher and Paykel, grants from Mutualia, grants and personal fees from Philips, grants and personal fees from Resmed, grants from Vitalaire, grants from Boehringer Ingelheim, grants fromJazz Pharmaceuticals, grants from Night Balance, and grants from Sefam, all outside the context of the submitted work.

## Authors’ contributions

 JLP: Conceptualization, supervision, funding acquisition, writing–review & editing. HR: Methodology, investigation, funding acquisition, writing–review & editing. PW: Methodology, investigation, data curation, writing–review & Editing. ND: Software, formal analysis, visualization, writing–original draft. SB: Software, formal analysis, visualization, writing–review & editing. AF: Interpretation, writing–original draft, writing–review & editing. RT: Conceptualization, supervision, writing–review & editing.

## Funding

 This work was supported by the French National Research Agency in the framework of the “Investissements d’avenir” program (ANR-15-IDEX-02) and the “e-health and integrated care and trajectories medicine MIAI artificial intelligence” chairs of excellence from the Grenoble Alpes University Foundation (JLP, HR, RT, and SB). AF was employed by Grenoble Alpes University Hospital. HR and PW were also supported by the French National Health Insurance (“Régime général de l’assurance maladie”). ND was supported by the national research and technology agency as part of a thesis grant (CIFRE) at Agir a dom (Healthcare provider). The funding sources had no involvement in the conduct of the research or preparation of the article.

## Supplementary files


Supplementary file 1. Additional Background Information.

Supplementary file 2. Additional Methods.

Supplementary file 3. Forgoing Healthcare Questionnaire.

Supplementary file 4. Additional Results.

Supplementary file 5. Additional Analysis and Discussion: Contextual Determinants of Forgoing Healthcare.
