# Supplementary material for: Why People Forgo Healthcare in France: A National Survey of 164 092 Individuals to Inform Healthcare Policy-Makers
Source: Int J Health Policy Manag. 2022 Jun 11;11(12):2972–81. doi: 10.34172/ijhpm.2022.6310 (PMC10105192; doi:10.34172/ijhpm.2022.6310)
Supplement: Supplementary file 1 — Additional Background Information. [file ijhpm-11-2972-s001.pdf]

Article title: Why People Forgo Healthcare in France: A National Survey of 164 092 Individuals to Inform Healthcare Policy-Makers

Journal name: International Journal of Health Policy and Management (IJHPM)

Authors' information: Najeh Daabek[1,2], Sébastien Bailly[1,3], Alison Foote[4], Philippe Warin[5], Renaud Tamisier[1,3], Hélèna Revil[5], Jean-Louis Pépin[1,3]*

[1]HP2 laboratory, INSERM U1300, University Grenoble Alpes, Grenoble, France.

[2]AGIR à Dom, Homecare charity, Grenoble, France.

[3]EFCR Laboratory, Grenoble Alpes University Hospital, Grenoble, France.

[4]Research Division, Grenoble Alpes University Hospital, Grenoble, France.

[5]Social Sciences Research – PACTE Laboratory, CNRS UMR 5194, University Grenoble Alpes, Grenoble, France.

(*Corresponding author: Email: jpepin@chu-grenoble.fr)

Supplementary file 1. Additional Background Information

Health insurance in France: Additional information about some physicians charging excess fees

In France, physicians (and more generally all health professionals) sign a contract with the compulsory health insurance (CNAM). They are then said to be "contracted". If they choose to practice in the contracted sector "1", they must respect the rates set at the national level between the governing bodies of their profession and the compulsory health insurance. In return, they benefit from a partial deduction of their social security contributions (illness, retirement, etc.). In contrast, physicians who contract to practice in the sector "2" ("non-contracted") do not benefit from these deductions but may charge higher fees. However, their patients are only reimbursed on the basis of the negotiated rates.
