# Supplementary material for: Why People Forgo Healthcare in France: A National Survey of 164 092 Individuals to Inform Healthcare Policy-Makers
Source: Int J Health Policy Manag. 2022 Jun 11;11(12):2972–81. doi: 10.34172/ijhpm.2022.6310 (PMC10105192; doi:10.34172/ijhpm.2022.6310)
Supplement: Supplementary file 2 — Additional Methods. [file ijhpm-11-2972-s002.pdf]

Article title: Why People Forgo Healthcare in France: A National Survey of 164 092 Individuals to Inform Healthcare Policy-Makers

Journal name: International Journal of Health Policy and Management (IJHPM)

Authors' information: Najeh Daabek[1,2], Sébastien Bailly[1,3], Alison Foote[4], Philippe Warin[5], Renaud Tamisier[1,3], Hélèna Revil[5], Jean-Louis Pépin[1,3]*

[1]HP2 laboratory, INSERM U1300, University Grenoble Alpes, Grenoble, France.

[2]AGIR à Dom, Homecare charity, Grenoble, France.

[3]EFCR Laboratory, Grenoble Alpes University Hospital, Grenoble, France.

[4]Research Division, Grenoble Alpes University Hospital, Grenoble, France.

[5]Social Sciences Research – PACTE Laboratory, CNRS UMR 5194, University Grenoble Alpes, Grenoble, France.

(*Corresponding author: Email: jpepin@chu-grenoble.fr)

Supplementary file 2. Additional Methods

Figure S1. BRS survey waves throughout France

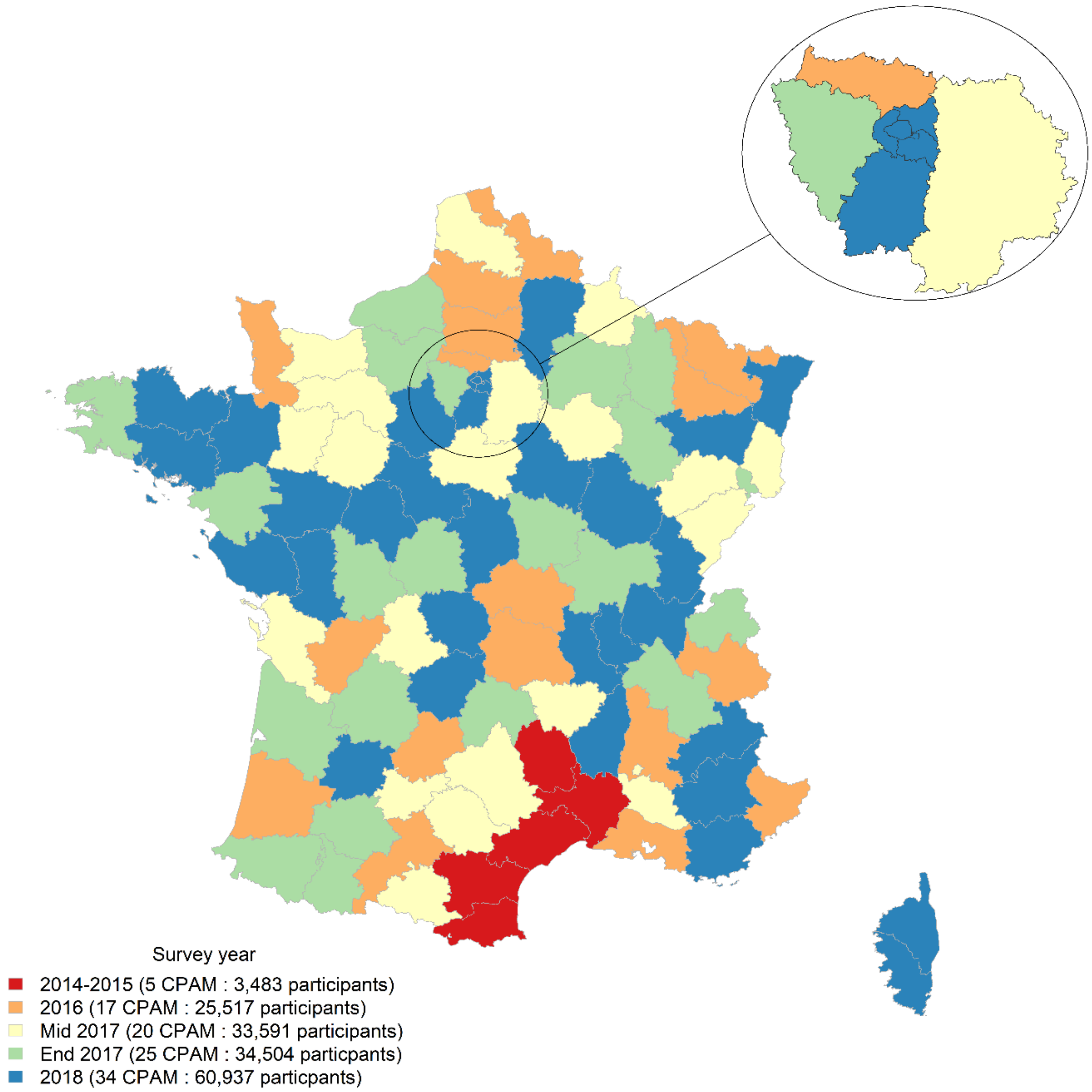

CPAM: Compulsory Primary Health Insurance agencies by “département” (county)
BRS: “Baromètre du renoncement aux soins” [Instrument to measure forgoing healthcare]
The inset shows the Paris agglomeration “Ile de France” region

Additional Methods 1: Spatial distribution of forgoing healthcare by county

The mean population-adjusted rate of forgoing healthcare was calculated for each county using a direct standardization method: First, the theoretical number of people forgoing healthcare was calculated by multiplying the raw rate from the questionnaires by the reference population, where the reference population was the number of people registered with the county's primary healthcare insurance agency (CPAM). Confounding factors used were age and sex. The population adjusted rate was then obtained by dividing the theoretical number of people forgoing healthcare by the total reference population.

Additional Methods 2: Details of Statistical methods

For each level (k-1) of the dependent variable, our final model can be written as:

$$\ln\left(\frac{\mathrm{P(k = Forwent\ healthcare\ for\ financial\ reasons)}}{\mathrm{P(k = Did\ not\ forgo\ healthcare)}}\right)$$
$$= b_{k0} + b_{k1}(\mathrm{Sex = Female}) + b_{k2}(\mathrm{Age = 25\ to\ 39\ years\ })$$
$$+ b_{k3}(\mathrm{Age = 25\ to\ 39\ years\ }) + b_{k4}(\mathrm{Age = 40\ to\ 59\ years\ })$$
$$+ b_{k5}(\mathrm{Age = \ More\ than\ 60\ years}) + \ b_{k6}(\mathrm{Family\ situation = \ Other\ })$$
$$+ b_{k7}(\mathrm{Family\ situation: \ = \ Alone\ })$$
$$+ b_{k8}(\mathrm{\ registered\ with\ a\ GP = \ Not\ registered\ \ })$$
$$+ b_{k9}(\mathrm{SPC\ and\ healthinsurance\ \ = \ Working\ (ACS)\ })$$
$$+ b_{k10}(\mathrm{SPC\ and\ healthinsurance\ \ = \ Not\ working\ (ACS)})$$
$$+ b_{k11}(\mathrm{SPC\ and\ healthinsurance\ \ = \ Retired\ (ACS)})$$
$$+ b_{k12}(\mathrm{SPC\ and\ healthinsurance\ \ = \ Working\ (CMU - C)})$$
$$+ b_{k13}(\mathrm{SPC\ and\ healthinsurance\ \ = \ Not\ working\ (CMU - C)})$$
$$+ b_{k14}(\mathrm{SPC\ and\ healthinsurance}$$
$$= \ \mathrm{Working\ with\ a\ private\ health\ insurance\ })$$
$$+ b_{k15}(\mathrm{SPC\ and\ healthinsurance}$$
$$= \ \mathrm{Not\ working\ with\ a\ private\ health\ insurance\ })$$
$$+ b_{k16}(\mathrm{SPC\ and\ healthinsurance}$$
$$= \ \mathrm{Retired\ with\ a\ private\ health\ insurance\ \ \ })$$
$$+ b_{k17}(\mathrm{SPC\ and\ healthinsurance}$$
$$= \ \mathrm{Not\ working\ without\ healthcare\ insurance})$$
$$+ b_{k18}(\mathrm{SPC\ and\ healthinsurance = Working\ without\ healthcare\ insurance})$$
$$+ b_{k19}(\mathrm{SPC\ and\ healthinsurance\ \ = \ Retired\ without\ a\ healthcare\ insurance\ })$$
$$+ \xi_i + \xi$$

Where:

ξ_i: Random effects ξ: error   SPC: Socio-professional category

Odds ratio interpretation: An example: using the sex variable:

- **Forgoing healthcare for financial reason:**

If $\mathbf{b_{k1}<1}$: The log odds of forgoing healthcare for financial reason vs. Did not forgo healthcare will decrease by $\mathbf{b_{k1}}$ if sex=female compared to sex=male.

If $\mathbf{b_{k1}>1}$: The log odds of forgoing healthcare for financial reason vs. Did not forgo healthcare will increase by $\mathbf{b_{k1}}$ if sex=female compared to sex=male.

If $\mathbf{b_{k1}=1}$: The log odds of forgoing healthcare for financial reason vs. Did not forgo healthcare does not change if sex=female compared to sex=male.

- **Forgoing healthcare for other reasons:**

If $\mathbf{b_{k1}<1}$: The log odds of forgoing healthcare for other reasons vs. Did not forgo healthcare will decrease by $\mathbf{b_{k1}}$ if sex=female compared to sex=male.

If $\mathbf{b_{k1}>1}$: The log odds of forgoing healthcare for other reasons vs. Did not forgo healthcare will increase by $\mathbf{b_{k1}}$ if sex=female compared to sex=male.

If $\mathbf{b_{k1}=1}$: The log odds of forgoing healthcare for other reasons vs. Did not forgo healthcare does not change if sex=female compared to sex=male.

ACS: [Aide à la complémentaire santé] = subsidized complementary health insurance scheme

CMU-C: [Couverture maladie universelle complémentaire] = complementary universal health insurance coverage
GP: General practitioner / primary care physician

Additional Methods 3: Statistical analysis; Combination categorical variables

The 12 modes of the categorical variable for the combination socio-professional category and complementary healthcare insurance were:

1. Working with public participation in private complementary health insurance (ACS)
2. Working with a public complementary health insurance (CMU-C)
3. Working without any complementary healthcare insurance
4. Working with a private complementary health insurance
5. Not-working with public participation in private complementary health insurance (ACS)
6. Not-working with a public private complementary health insurance (CMU-C)
7. Not-working without private complementary insurance
8. Not-working with a private complementary health insurance
9. Retired with a public participation in private complementary health insurance (ACS)
10. Retired with a public private complementary health insurance (CMU-C)
11. Retired without a private complementary healthcare insurance
12. Retired with a private complementary health insurance

ACS: [Aide à la complémentaire santé] = subsidized complementary health insurance scheme

CMU: [Couverture maladie universelle complémentaire] = complementary universal health insurance coverage
