# Supplementary material for: Why People Forgo Healthcare in France: A National Survey of 164 092 Individuals to Inform Healthcare Policy-Makers
Source: Int J Health Policy Manag. 2022 Jun 11;11(12):2972–81. doi: 10.34172/ijhpm.2022.6310 (PMC10105192; doi:10.34172/ijhpm.2022.6310)
Supplement: Supplementary file 3 — Forgoing Healthcare Questionnaire. [file ijhpm-11-2972-s003.pdf]

Article title: Why People Forgo Healthcare in France: A National Survey of 164 092 Individuals to Inform Healthcare Policy-Makers

Journal name: International Journal of Health Policy and Management (IJHPM)

Authors' information: Najeh Daabek[1,2], Sébastien Bailly[1,3], Alison Foote[4], Philippe Warin[5], Renaud Tamisier[1,3], Hélèna Revil[5], Jean-Louis Pépin[1,3]*

[1]HP2 laboratory, INSERM U1300, University Grenoble Alpes, Grenoble, France.

[2]AGIR à Dom, Homecare charity, Grenoble, France.

[3]EFCR Laboratory, Grenoble Alpes University Hospital, Grenoble, France.

[4]Research Division, Grenoble Alpes University Hospital, Grenoble, France.

[5]Social Sciences Research – PACTE Laboratory, CNRS UMR 5194, University Grenoble Alpes, Grenoble, France.

(*Corresponding author: Email: jpepin@chu-grenoble.fr)

Supplementary file 3. Additional Methods

Supplementary file 3: Forgoing Healthcare questionnaire (BRS)

(Translated from the original French version used by the CPAM agencies)

HEALTH CARE SITUATION TRACKING

-RESERVED FOR INTERVIEWING AGENT-

1. Your County: ....

2. Your function:

CPAM
- ◯ Reception Agent
- ◯ ASS Agent
- ◯ CES Agent
- ◯ Prado Agent

Medical service
- ◯ Administrative Agent
- ◯ Medical advisor

- ◯ CARSAT Agent
- ◯ CCAS Agent

- ◯ CHRUS Agent
- ◯ MSA Agent

- ◯ UDAF Agent
- ◯ Médecins du monde

-QUESTIONS TO THE INTERVIEWEE -

3. ◯ General ◯ MSA ◯ RSI ◯ Other

4. Postal code : ....

5. Sex: ◯ Male ◯ Female

6. Age :
- ◯ Under 18 years
- ◯ 18-24 years
- ◯ 25-39 years
- ◯ 40-59 years
- ◯ Over 60 years

7. Family situation
- ◯ Living alone
- ◯ Alone with children
- ◯ Childless couple
- ◯ Couple with children
- ◯ Other *(seniors living with their children)*

8. Socio-professional category
- ◯ Working farmer
- ◯ Artisan, merchant
- ◯ Manager
- ◯ Intermediate professions
- ◯ Employee
- ◯ Manual worker
- ◯ Retired
- ◯ Unemployed

9. Do you have a complementary Health insurance?
- ◯ Yes, the CMU-c
- ◯ Yes, ACS
- ◯ Yes, another insurance
- ◯ Yes, the AME
- ◯ No
- ◯ Don't know

10. If you don't have a complementary Health insurance, why?
- ◯ It would not help, I'm never sick
- ◯ I have other financial priorities
- ◯ I haven't the means
- ◯ I don't know
- ◯ I lack information
- ◯ By negligence
- ◯ I benefited from a complementary company but no longer
- ◯ I'm covered at 100% by my primary insurance
- ◯ Anyway, I will be badly reimbursed
- ◯ The steps are too complicated
- ◯ I do not know what purpose it serves
- ◯ Other, please give details: ....

....

11. Do you have a referring physician/ general practitioner?

◯ Yes ◯ No ◯ Don't know

12. When was your last visit to a doctor?
- ◯ In the last 6 months
- ◯ Between 6 months and 1 year ago
- ◯ Between 1 and 2 years ago
- ◯ Don't know
- ◯ More than 2 years ago

13. How do you perceive your overall health?
- ◯ Very good
- ◯ Good
- ◯ Average
- ◯ Poor
- ◯ Very poor

14. Have you forgone or put-off healthcare on one or more occasions in the last 12 months?

◯ Yes ◯ No

If no, skip to question 21

15. Since when have you been forgoing healthcare?
- ◯ Less than 6 months
- ◯ Between 6 months and 1 year
- ◯ Between 1 year and 2 years
- ◯ More than 5 years
- ◯ Between 2 years and 5 years

16. Do you think this situation could change in the near future?

◯ Yes ◯ No ◯ Don't know

17. In general, do you feel that you can get care when you need it?

◯ Yes ◯ No ◯ Don't know

18. What types of care have you forgone? *(7 answers maximum)*

- ◯ Surgery
- ◯ Consultations with a primary care physician
- ◯ Consultations with a gynecologist
- ◯ Consultations with a dermatologist
- ◯ Consultations with a psychiatrist
- ◯ Consultations with an ophthalmologist
- ◯ Consultations with a cardiologist
- ◯ Consultations with another specialist: ……………………
- ◯ Restorative dental care (caries, descaling, infections…)
- ◯ Prosthetic dental care (implants, bridges…)
- ◯ Orthodontic care
- ◯ Physical Therapy
- ◯ Hearing device
- ◯ Medical analyses
- ◯ Adapted glasses or lenses
- ◯ Purchase of medication
- ◯ Prescribed work stoppage
- ◯ Speech and language therapy
- ◯ Other: ……………………………………………………

19. Reasons for forgoing healthcare? *(5 answers maximum)*

- ◯ Self-pay part of cost too expensive
- ◯ Geographical distance
- ◯ Non-urgent care
- ◯ Work related fears (stress, work stoppage…)
- ◯ Care refusal by practitioner (saturation, discrimination)
- ◯ Expensive transport costs
- ◯ Availability (lack of time)
- ◯ Do not know a practitioner
- ◯ Loss of income due to work stoppage
- ◯ Absence of means of transport
- ◯ Lassitude
- ◯ Delays to get an appointment too long
- ◯ Cannot advance costs
- ◯ Physical inability to visit a physician
- ◯ Negligence
- ◯ Fear of the doctor
- ◯ Fear of diagnosis
- ◯ I do not know how much I will be asked to pay
- ◯ The steps are too complicated (need for accompaniment)
- ◯ Lack of information about the system
- ◯ Other: …………………………………………………

20. Do you feel that this situation of forgoing healthcare will have consequences? *(3 answers maximum)*

- ◯ Health care consequences
- ◯ Professional life consequences
- ◯ Social life consequences
- ◯ Family life consequences
- ◯ Ability to continue living at home
- ◯ Other:
- ◯ ……………………………………………………

-QUESTIONS ABOUT THE INTERVIEWEE'S FAMILY ENVIRONMENT-

21. Has a family member forgone or put off care? ◯ Ye ◯ No

22. If so, who was it? *(2 answers*

- ◯ Your spouse
- ◯ Your child/children
- ◯ Another person, to be defined: ……………………………………

23. What are the types of care forgone? *(7 answers maximum)*

- ◯ Surgery
- ◯ Consultations with a primary care physician
- ◯ Consultations with a gynecologist
- ◯ Consultations with a dermatologist
- ◯ Consultations with a psychiatrist
- ◯ Consultations with an ophthalmologist
- ◯ Consultations with a cardiologist
- ◯ Consultations with another specialist: ……………………
- ◯ Restorative dental care (caries, descaling, infections…)
- ◯ Prosthetic dental care (implants, bridges…)
- ◯ Orthodontic care
- ◯ Physical Therapy
- ◯ Hearing device
- ◯ Further examinations
- ◯ Glasses or contact lenses
- ◯ Therapy
- ◯ Prescribed leave of absence
- ◯ Speech and language therapy
- ◯ Other: ……………………………………………………

24. Do you agree to be interviwed by the person in charge of the study? *(Only applicable to people forgoing*

- ◯ Yes
  - ☐ At the CPAM
  - ☐ At home
- ◯ No

Abbreviations in Questionnaire

	**French**	**English**
CPAM	Caisse Primaire d’assurance Maladie	Primary Health Insurance scheme (compulsory)
CSS	Complément santé solidaire	
CARSAT	Caisse d'Assurance Retraite et de la Santé au Travail Rhône-Alpes	Agency for Pensions and Health at work
CCAS	Centre Communal d'Action Sociale	
CCSS	Caisse commune de sécurité sociale (CCSS)	
CHRUS	Centre Hospitalier Régionale	Regional Hospital
MSA	Mutualité sociale agricole	Agricultural workers’ insurance scheme (compulsory)
RSI	Régime social des Indépendants	Social security scheme for self-employed workers
UDAF	Union Départementale des Associations Familiales	Regional union of family associations
