# Supplementary material for: Why People Forgo Healthcare in France: A National Survey of 164 092 Individuals to Inform Healthcare Policy-Makers
Source: Int J Health Policy Manag. 2022 Jun 11;11(12):2972–81. doi: 10.34172/ijhpm.2022.6310 (PMC10105192; doi:10.34172/ijhpm.2022.6310)
Supplement: Supplementary file 4 — Additional Results. [file ijhpm-11-2972-s004.pdf]

Article title: Why People Forgo Healthcare in France: A National Survey of 164 092 Individuals to Inform Healthcare Policy-Makers

Journal name: International Journal of Health Policy and Management (IJHPM)

Authors' information: Najeh Daabek[1,2], Sébastien Bailly[1,3], Alison Foote[4], Philippe Warin[5], Renaud Tamisier[1,3], Hélèna Revil[5], Jean-Louis Pépin[1,3]*

[1]HP2 laboratory, INSERM U1300, University Grenoble Alpes, Grenoble, France.

[2]AGIR à Dom, Homecare charity, Grenoble, France.

[3]EFCR Laboratory, Grenoble Alpes University Hospital, Grenoble, France.

[4]Research Division, Grenoble Alpes University Hospital, Grenoble, France.

[5]Social Sciences Research – PACTE Laboratory, CNRS UMR 5194, University Grenoble Alpes, Grenoble, France.

(*Corresponding author: Email: jpepin@chu-grenoble.fr)

Supplementary file 4. Additional Results

Figure S2: Study population

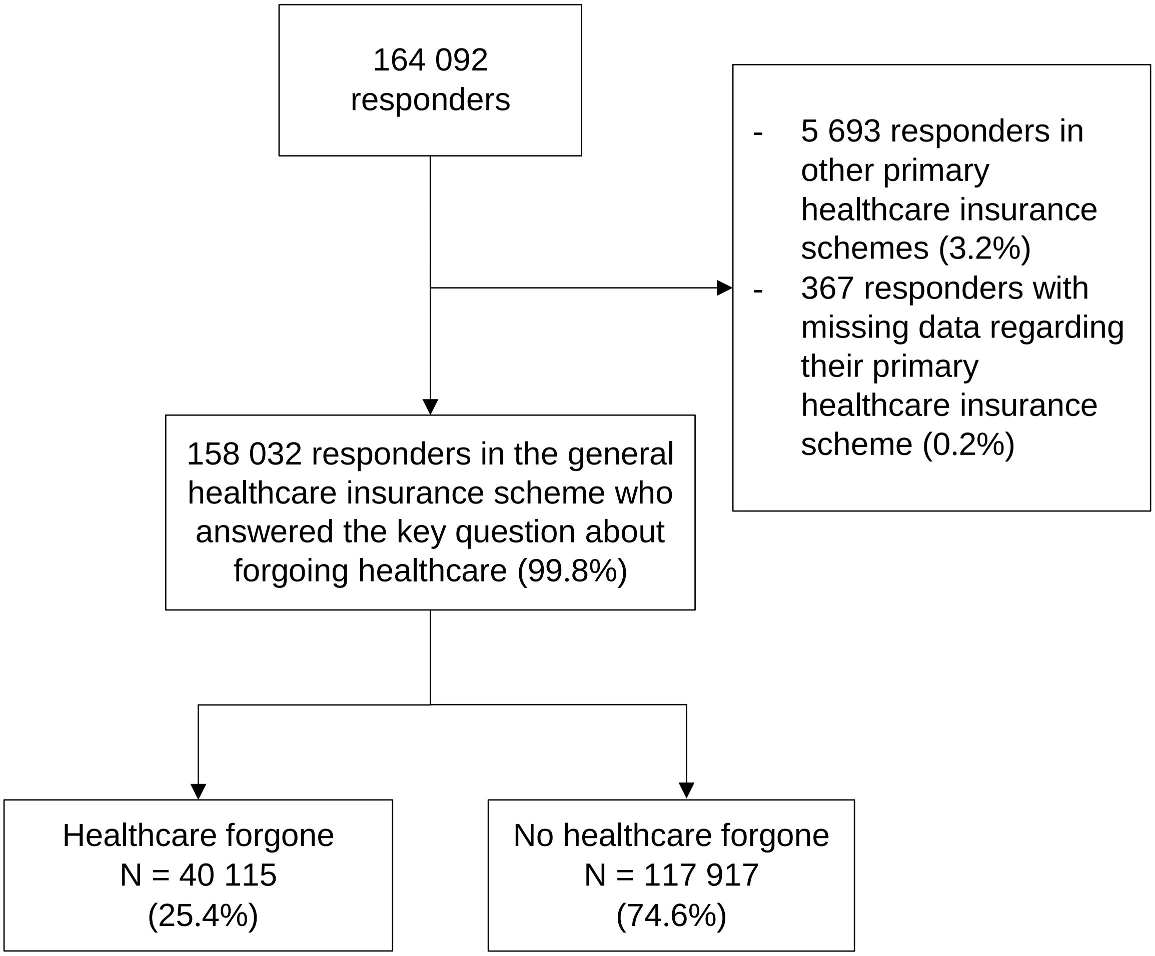

No register was kept of individuals who refused to fill-in the questionnaire.

Table S1: Alternative subgroup analysis “working” vs “retired/not-working” groups

Subgroup analysis:

a. Workers (N=76,671)

Workers group included intermediate professions, artisans, merchants, working farmers, managers and employees

Variable	Outcome	OR		
**Sex**				<.0001
Female vs Male	No healthcare forgone			
	Healthcare forgone because of financial issues	1.54	[1.47;1.60]	
	Healthcare forgone because of other issues	1.36	[1.289;1.44]	
**Age**				<.0001
25 to 39 years vs Under 24 years	No healthcare forgone			
	Healthcare forgone because of financial issues	1.35	[1.23;1.48]	
	Healthcare forgone because of other issues	1.13	[1.02;1.26]	
40 to 59 years vs Under 24 years	No healthcare forgone			
	Healthcare forgone because of financial issues	1.55	[1.42;1.69]	
	Healthcare forgone because of other issues	1.03	[0.93;1.15]	
Over 60 years vs Under 24 years	No healthcare forgone			
	Healthcare forgone because of financial issues	1.66	[1.48;1.87]	
	Healthcare forgone because of other issues	0.84	[0.72;0.99]	
**Family situation**				<.0001
Other vs Couple without children	No healthcare forgone			
	Healthcare forgone because of financial issues	1.37	[1.12;1.67]	
	Healthcare forgone because of other issues	1.31	[1.038;1.67]	
Couple with children vs Couple without children	No healthcare forgone			

Variable	Outcome	OR		
	Healthcare forgone because of financial issues	1.33	[1.25;1.41]	
	Healthcare forgone because of other issues	1.19	[1.11;1.29]	
Living alone vs Couple without children	No healthcare forgone			
	Healthcare forgone because of financial issues	1.49	[1.40;1.59]	
	Healthcare forgone because of other issues	1.09	[1.005;1.18]	
Living alone with children vs Couple without children	No healthcare forgone			
	Healthcare forgone because of financial issues	2.14	[1.99;2.29]	
	Healthcare forgone because of other issues	1.41	[1.28;1.55]	
Referring physician				<.0001
Not registered with a GP vs Registered with a GP	No healthcare forgone			
	Healthcare forgone because of financial issues	1.15	[1.03;1.28]	
	Healthcare forgone because of other issues	1.81	[1.60;2.05]	
Complementary healthcare insurance				<.0001
ACS vs CMU-C	No healthcare forgone			
	Healthcare forgone because of financial issues	1.95	[1.75;2.17]	
	Healthcare forgone because of other issues	0.70	[0.60;0.82]	
Without complementary health insurance vs CMU-C	No healthcare forgone			
	Healthcare forgone because of financial issues	3.97	[3.64;4.34]	
	Healthcare forgone because of other issues	1.16	[1.03;1.32]	
Private complementary health insurance vs CMU-C	No healthcare forgone			
	Healthcare forgone because of financial issues	1.44	[1.34;1.54]	
	Healthcare forgone because of other issues	0.89	[0.82;0.96]	

b. Non-Workers and the retired (N=81,361)

The non-working group included retired and unemployed

**Variable**	**Outcome**	**OR**		
**Sex**				<.0001
Female vs Male	No healthcare forgone			
	Healthcare forgone because of financial issues	1.38	[1.32;1.43]	
	Healthcare forgone because of other issues	1.35	[1.27;1.43]	
Age				<.0001
25 to 39 years vs Under 24 years	No healthcare forgone			
	Healthcare forgone because of financial issues	1.49	[1.37;1.61]	
	Healthcare forgone because of other issues	1.09	[0.99;1.21]	
40 to 59 years vs Under 24 years	No healthcare forgone			
	Healthcare forgone because of financial issues	2.02	[1.88;2.18]	
	Healthcare forgone because of other issues	1.17	[1.07;1.29]	
Over 60 years vs Under 24 years	No healthcare forgone			
	Healthcare forgone because of financial issues	1.24	[1.14;1.34]	
	Healthcare forgone because of other issues	0.69	[0.63;0.77]	
**Family situation**				<.0001
Other vs Couple without children	No healthcare forgone			
	Healthcare forgone because of financial issues	1.15	[1.00;1.31]	
	Healthcare forgone because of other issues	1.34	[1.13;1.58]	
Couple with children vs Couple without children	No healthcare forgone			
	Healthcare forgone because of financial issues	1.32	[1.24;1.41]	
	Healthcare forgone because of other issues	1.32	[1.20;1.46]	
Living alone vs Couple without children	No healthcare forgone			

Variable	Outcome	OR		
	Healthcare forgone because of financial issues	1.57	[1.49;1.66]	
	Healthcare forgone because of other issues	1.36	[1.26;1.48]	
Living alone with children vs Couple without children	No healthcare forgone			
	Healthcare forgone because of financial issues	1.76	[1.65;1.90]	
	Healthcare forgone because of other issues	1.50	[1.35;1.66]	
**Referring physician**				<.0001
Not registered with a GP vs Registered with a GP	No healthcare forgone			
	Healthcare forgone because of financial issues	1.08	[1.003;1.16]	
	Healthcare forgone because of other issues	1.51	[1.37;1.66]	
**Complementary healthcare insurance**				<.0001
ACS vs CMU-C	No healthcare forgone			
	Healthcare forgone because of financial issues	2.06	[1.92;2.20]	
	Healthcare forgone because of other issues	0.75	[0.68;0.83]	
Without complementary health insurance vs CMU-C	No healthcare forgone			
	Healthcare forgone because of financial issues	4.21	[3.98;4.46]	
	Healthcare forgone because of other issues	1.08	[1.00;1.18]	
Private complementary health insurance vs CMU-C	No healthcare forgone			
	Healthcare forgone because of financial issues	1.61	[1.53;1.70]	
	Healthcare forgone because of other issues	0.78	[0.73;0.84]	

ACS: [Aide à la complémentaire santé] = subsidized complementary health insurance scheme

CMU-C: (Couverture maladie universelle complémentaire] = complementary universal health insurance coverage
GP: General practitioner / primary care physician

Table S3: Effect of household composition: with and without children

	**Total sample N=158,032** N(%)	**Not forgoing Care N=117,917 (74.6%)** N(%)	**Forgoing Care N=40,115 (25.4%)** N(%)	Missing (%)	p-value*
**Family situation**					
Other	3345 (2.1)	2585 (2.2)	760 (1.9)	0.23	<.0001
Living as a couple with children	41346 (26.2)	31052 (26.4)	10294 (25.7)	.	.
Living as a couple	35054 (22.2)	28195 (24)	6859 (17.1)	.	.
Living alone	56831 (36)	41680 (35.4)	15151 (37.9)	.	.
Living alone with children	21085 (13.4)	14139 (12)	6946 (17.4)	.	
