# Supplementary material for: Why People Forgo Healthcare in France: A National Survey of 164 092 Individuals to Inform Healthcare Policy-Makers
Source: Int J Health Policy Manag. 2022 Jun 11;11(12):2972–81. doi: 10.34172/ijhpm.2022.6310 (PMC10105192; doi:10.34172/ijhpm.2022.6310)
Supplement: Supplementary file 5 — Additional Analysis and Discussion: Contextual Determinants of Forgoing Healthcare. [file ijhpm-11-2972-s005.pdf]

Article title: Why People Forgo Healthcare in France: A National Survey of 164 092 Individuals to Inform Healthcare Policy-Makers

Journal name: International Journal of Health Policy and Management (IJHPM)

Authors' information: Najeh Daabek[1,2], Sébastien Bailly[1,3], Alison Foote[4], Philippe Warin[5], Renaud Tamisier[1,3], Hélèna Revil[5], Jean-Louis Pépin[1,3]*

[1]HP2 laboratory, INSERM U1300, University Grenoble Alpes, Grenoble, France.

[2]AGIR à Dom, Homecare charity, Grenoble, France.

[3]EFCR Laboratory, Grenoble Alpes University Hospital, Grenoble, France.

[4]Research Division, Grenoble Alpes University Hospital, Grenoble, France.

[5]Social Sciences Research – PACTE Laboratory, CNRS UMR 5194, University Grenoble Alpes, Grenoble, France.

(*Corresponding author: Email: jpepin@chu-grenoble.fr)

Supplementary file 5. Additional Analysis and Discussion: Contextual Determinants of Forgoing Healthcare

Contextual data for each geographical unit were provided by the French National Institute for Statistic and Economic Studies (INSEE) (https://www.insee.fr/fr/accueil). These included socioeconomic data (median standard of living and poverty index per county), demographic factors (urbanized and rural counties) and accessibility factors (total number of physicians (general practitioners, specialists in private practice and hospital physicians) per 100,000 inhabitants.

We explored whether there were any associations between the county's population-adjusted mean rate of forgoing healthcare, and contextual socioeconomic, demographic and accessibility factors using a linear spatial autoregressive model (SAR) [37] and a Durbin model [38] for each of the contextual variables. The quality of the models was assessed using R squared values and Moran's index of residuals.

The association between the population-adjusted mean rate of forgoing healthcare and each of the contextual determinants explored using two spatial models (SAR and Durbin) gave poor quality models and no conclusive results.

Limitations to contextual determinants analysis and to the main study

We were not able to draw any conclusions concerning the association between contextual determinants and the probability of forgoing healthcare due to the poor quality of our models.

This might be explained by the choice of the French administrative area known as a “département” (equivalent in size to a county in the US or GB and hereafter called a county) as our spatial unit of analysis. This was because CPAM is organized on a county-wide basis, irrespective of the demographic distribution within the county, whereas the French census body, INSEE, uses a smaller spatial unit, zones, which better reflect population density, density of healthcare providers etc. Given the often wide variability of population density and characteristics etc. within a county the analysis of contextual effects needed a smaller spatial unit. Moreover, the definitions we used of an urbanized county as being one in which >50% of individuals live in an urban zone, and of a rural county as being one in which >50% of individuals live in rural zone seems rather biased in favor of ‘urban’ counties, without any account being taken of the influence of large urban agglomerations.

Furthermore, our ‘rural’ population was misrepresentative because the survey was only given to people registered with the general scheme (CPAM) and not individuals (and their families) registered in the compulsory primary healthcare insurance scheme for agricultural workers (Departmental fund of the Agricultural Mutual Assistance Association).

We used socio-professional category rather than socio-economic category, which can be very different, because INSEE collects socio-professional category and not socio-economic category rendering economic analyses more difficult. While household income can be requested this is a rather sensitive issue, and income tax-band is confidential, as are family allowance data. In France one is not allowed by law to collect data on ethnic origin.

References for Supplementary file 5:

[37] Hoef JMV, Peterson EE, Hooten MB, Hanks EM, Fortin M-J. Spatial autoregressive models for statistical inference from ecological data. Ecological Monographs. 2018;88(1):36-59. doi:10.1002/ecm.1283

[38] Feng Y, Wang X, Du W, Liu J. Effects of Air Pollution Control on Urban Development Quality in Chinese Cities Based on Spatial Durbin Model. International Journal of Environmental Research and Public Health. 2018;15(12):2822. doi:10.3390/ijerph15122822
